# A Rolling Bearing Fault Diagnosis Based on Conditional Depth Convolution Countermeasure Generation Networks under Small Samples

**DOI:** 10.3390/s22155658

**Published:** 2022-07-28

**Authors:** Cheng Peng, Shuting Zhang, Changyun Li

**Affiliations:** 1 School of Computer, Hunan University of Technology, Zhuzhou 412007, China; pengcheng@hut.edu.cn (C.P.); zhangst06@163.com (S.Z.); 2 School of Automation, Central South University, Changsha 410083, China

**Keywords:** small sample, data augmentation, spectral normalization, fault diagnosis

## Abstract

Aiming at the problems of low fault diagnosis accuracy caused by insufficient samples and unbalanced data sample distribution in bearing fault diagnosis, this paper proposes a fault diagnosis method for rolling bearings referencing conditional deep convolution adversarial generative networks (C−DCGAN) for efficient data augmentation. Firstly, the concept of conditional constraints is used to guide and improve the sample generation process of the original generative adversarial network, and specific constraints are added to the data generation model to perform a balanced expansion of muti-category fault data for small sample data sets. Secondly, aiming at the phenomena of training instability, gradient disappearance and gradient explosion in the imbalanced sample set, it is proposed to optimize the structure of the generative network by using the structure of self-defined skip connections and spectral normalization, while using the Wasserstein distance with penalty term instead of cross entropy. The function is used as the loss function of the generative adversarial network to improve the stable feature extraction ability of the generative network and the effect of the training process; in this way, simulation sample data with only a small variation from the real data distribution can be generated. Finally, the complete fault data set (after mixing the original data with sufficient fault category and sample number) and the generated data are input into the one-dimensional convolution neural network for fault diagnosis of rolling bearing. The experiment’s results show that the diagnosis method in this paper can improve the fault classification effect of rolling bearings by generating balanced and sufficient sample data.

## 1. Introduction

Mechanical equipment is composed of many parts, and the bearing is an important part of heavy industry; over the long-term operation of mechanical equipment, the bearings may often be easily damaged. In daily operation, when the mechanical failure of bearings occurs, it risks accidents, even ones resulting in losses to factories and enterprises [1,2,3]. Therefore, if a specific fault diagnosis is made for a rolling bearing in the process of operation, the fault types of the bearing can be identified in time, and the faults of the bearing can be repaired or replaced to different degrees; such precautions can effectively ensure the operation of the mechanical equipment, and avoid accidents [4,5].

In terms of industrial machinery fault diagnosis, there are mainly two types of fault diagnosis and detection methods. The first is to use the model to compare the output value of the model with the actual signal data during the operation of the machine, so as to distinguish different potential failures caused by the machine, and to infer and classify these predictions according to the failure phenomenon. The second is a data-driven fault diagnosis method, which usually collects a large number of vibration signals during the actual production process when the machinery is running, describing the state information of the machinery operation, and then performs feature learning from the data to classify the faults. The deep learning model has powerful feature extraction capabilities based on its own structural characteristics, and can autonomously extract hidden features, which can be used to deal with a large number of complex fault features. Therefore, in recent years, deep learning-based fault diagnosis methods have been highly applicable to the field of faults diagnostics. Common methods used are auto-encoder (AE) [6], long-short term memory network (LSTM) [7] and Convolutional Neural Network (CNN) [8]. CNN is an artificial neural network based on the connection of multiple convolutional layers. Because the design of its frame structure uses the convolution kernel in the convolutional layer to automatically extract hidden features from sample data, CNN is mainly suitable for use in the fields of model recognition and fault diagnosis and detection [9,10,11]. However, the above three common fault diagnosis methods are all supervised learning methods, and their training process requires a large amount of effective sample data [12]. Due to the lack of fault data in the actual operation of mechanical equipment and the fact that the majority of data collected is normal data, the collected bearing fault data set risks the hazards of insufficient or unbalanced sample data [13,14]. Generally, previous efforts have been unable to train deep learning models with high classification accuracy. In the process of testing, the model ignores some fault categories with less sample data, and as a result, the fault cannot be recognized in time [15]. In view of the problem of small sample data which is therefore unbalanced and insufficient, past efforts have mainly used the following several solutions. 

The first method is statistical learning theory [16]. The theory uses the small sample method of machine learning to enhance data, but it has some disadvantages, such as poor generalization and slow computation. The second type of method is image enhancement [17,18]. The basic idea of this method is to adjust and transform the data set of small scale to increase the number of samples, and improve the sample diversity for example, the image is switched to intercept and get different sample images. Although this method has good generalization capacities and can be widely used in the study of one-dimensional or two-dimensional small sample problems, because this method is based on the modification of original sample data, it is easy to cause the data distribution to be too unitary, and the sample repetition rate is high. The third kind of method is the Generative Adversarial Networks (GAN) [19]; the generation structure module and the discriminative structure module in the network are used to generate sample data through adversarial gameplaying in order to solve the problems of insufficient samples or unbalanced samples [20].

For the third type of method, Liu et al. [21] put forward a data set enhancement algorithm which is based on the original sample set. This algorithm divides the original sample data into groups based on several special monomers, and makes use of the combination of different monomers to change the data, thereby enhancing the data from small samples; these changes can be verified by subsequent experiments to improve the performance of learning data features and classification. Meng et al. [22] proposed a rotating machinery fault diagnosis framework based on GANs and multi-sensor data fusion. Xue and others [23] proposed a diagnosis algorithm for sample set enhancement using Wasserstein Generative Adversarial Networks (WGAN), which balanced the data distribution of small sample data sets through WGAN to solve the problem of poor generalization in the training process of fault diagnosis caused by serious imbalances of data. Radford et al. [24] proposed a new generation network, namely the deep convolution generative adversarial networks (DCGAN). On the basis of GAN, the step-size convolution is used to improve both the stability of the network training process and the validity of the generated data. However, DCCAN can only be used for small scale data generation, not for large scale sample data enhancement. Gauthier et al. [25] proposed conditional generative adversarial networks (CGAN), which synchronously inputted the input value and the specific constraint into the network structure and generated the sample data for the specific constraint. However, the problem of instability of the training process in the original GAN cannot be solved. Gao et al. [26] proposed to combine Wasserstein generative adversarial nets (WGAN) and convolutional neural network to construct WG−CNN, so as to carry out bearing fault diagnosis through data generation.

In summary, this paper proposes a bearing fault diagnosis method based on conditional deep convolution generative adversarial networks (C-DCGAN) on the basis of DCGAN and extra constraint condition *C*. The problem of the unbalanced distribution of generated data is solved by defining constraint condition *C* which is classification labels to constrain the generating process. According to the experimental results from the Case Western Reserve University (CWRU) Bearing Data Center, this method can effectively enhance muti-category data from small sample sets and improve the accuracy of bearing fault diagnosis while increasing the training stability of the generating network.

## 2. Related Works

### 2.1. DCGAN

A GAN consists of a generator and a discriminator. Random data is used as the input value of the generator, and the output G (*z*) and the real sample value are input into the discriminator at the same time to discriminate the generated data. Through the game between the two, the difference between the data distribution of the generated sample and that of the real sample is gradually reduced to a certain extent, which then permits the inference that the generation model has trained convergence; the generator in the model can then be used to generate data which will be similar to the data distribution of real sample data.

The differences between DCGAN and GAN are that: (1) in the discriminator of DCGAN, the pool layer is removed and the convolution layer is added to the structure; (2) by adding batch normalization, the problem that all fault samples converge to the same point is solved; (3) all the fully connected layers in the GAN structure are removed and replaced by convolution layers; (4) the activation functions of each layer of the DCGAN generator and discriminator are modified and replaced, and the instability and gradient disappearance in the original GAN are solved by using the characteristics and definitions of different activation functions. DCGAN essentially maximizes the discriminator’s accuracy with respect to the data source of the input value and minimizes the difference between the generator’s output and the real sample. This game process can be seen as a maximization and minimization problem, expressed in Formula (1):

(1) $$\underset{G}{\min}\;\underset{D}{\max}\;V(G,D)=E_{x\sim P_{data}(x)}[\log D(x)]+E_{z\sim P_{z}(z)}[\log(1-D(G(z)))]$$

Among them, *P_data_* is the data distribution of the real sample value, and *P_z_* is the data distribution of the random noise 

### 2.2. CGAN

In 2014, based on GAN, condition *C* was added to each input of its network structure, and a conditional generative adversarial network (CGAN) was proposed [27]. Condition *C* can be any kind of auxiliary information, such as a specific set of sample input values.

Taking random noise *Z* and constraint *C* as input conditions, sample *G* (*Z*|*C*) is generated by the generator. According to constraint *C*, the discriminator can distinguish whether the input value is the real sample or not. The objective function *V* (*D*, *G*) is:

(2) $$\underset{G}{\min}\;\underset{D}{\max}\;V(D,G)=E_{x\sim p_{data(x)}^{}}[\log D(x|c)]+E_{x\sim P_{z(z)}}[\log(1-D(G(z|c)))]$$

CGAN makes use of the constraint condition *C* to constrain the training process of the generating model, and outputs *G*(*Z*|*C*), which conforms to the specific constraint condition, while the discriminator uses the real sample *x* and the specific constraint condition *C*, which is the probability of discriminating the input value as the real sample value.

## 3. C-DCGAN for Bearing Fault Diagnosis

In this paper, a C-DCGAN network structure is proposed, in which the bearing fault category label is defined as specific constraint *C*. The fault category label is processed by one-hot encoding, and the one-bit valid code of the original label of the fault data is then defined as the constraint *C* in this paper. There are 10 categories in total, and the generated data can be restricted. Addressing the problems of training instability, gradient disappearance and gradient explosion of the original network model, solutions are summarized as follows:

(1) Using custom residual blocks, the regularization effect of the generating network is optimized, the feature extraction ability of the network is enhanced, and the authenticity of the generated simulation fault data is improved.
(2) In the generator and discriminator, spectral normalization is added to improve the stability of the network model training in this experiment, and to solve the gradient explosion problem.
(3) The loss function is set as *W* distance with penalty term, which effectively solves the problem of gradient disappearance.

### 3.1. Conditional Deep Convolution Antagonism Generation Network Structure

In this paper, C−DCGAN uses the iterative training process to study the relation between the constraint condition *C* and the mapping relationship between the random noise and the original fault sample data, and also to supervise the generator whether in accordance with condition *C*; the problem of uneven distribution and insufficient number of bearing fault samples is solved by generating bearing fault data which obey the distribution of real fault samples.

**Figure 1:**
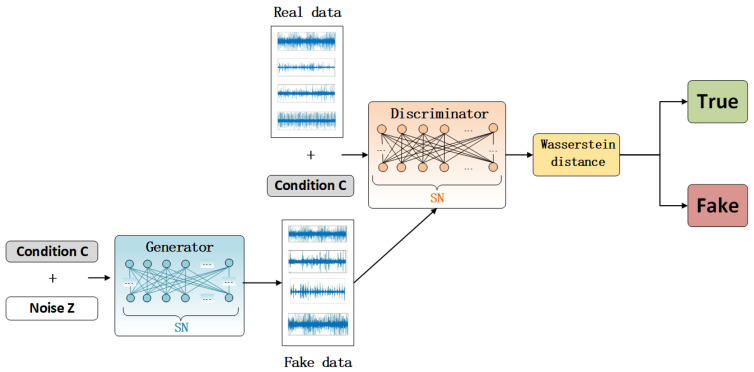
Conditional Deep Convolution Generative Adversarial Networks.

The structure of the C−DCGAN network is shown in Figure 1.

In C-DCGAN, the objective function is:

(3) $$\underset{G}{\min}\;\underset{D}{\max}\;V(D,G)=E_{x\sim p_{data(x)}^{}}[\log D(x)]+E_{x\sim P_{z(z)}}[\log(1-D(G(z|c)))]$$

*E* (·) is the expectation of the objective function, the distribution of the real fault data is *P*_data_, and the distribution of the input noise data is *P_z_*.

**Figure 2:**
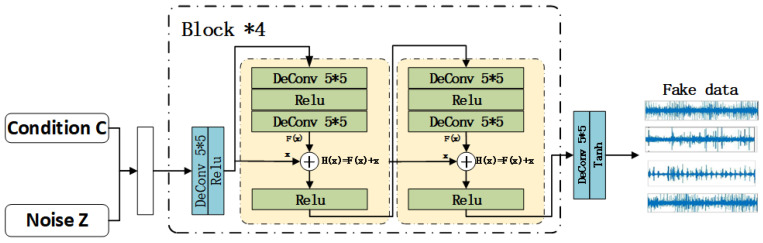
C-DCGAN generator.

In the generator, the constraint condition *C* and the random noise *Z* are input into the generator synchronously as input values, and the five transposed convolution layers are connected to each other; between each transposed convolution layer, the network structure is deepened by the residual structure [28], that is, the transposed convolution layer, the activation function layer, the transposed convolution layer, the summation operation *h*(*x*) and the activation function layer are superposed one by one to form a self-defined residual block; using the jump connection in the residual block, the mapping *F*(*x*) and the input value *x* are superimposed for output to reduce additional calculations and parameter values. In the output layer, the Tanh function functions as the activation function, which improves the convergence speed and reduces the number of iterations, while in the input layer and other intermediate layers, the Relu function is chosen as the activation function, and the calculation process is thereby simplified and the problem of gradient disappearance is solved. The network structure deepened in this experiment is only composed of two residual blocks, because excessive network depth may lead to problems such as unstable training and loss of data features, as shown in Figure 2.

**Figure 3:**
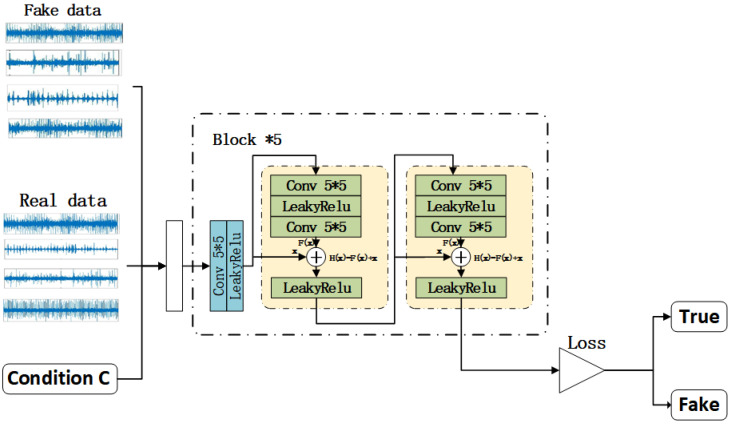
C-DCGAN discriminator.

In the discriminator, the constraint condition *C*, the generator output *G*(*Z*|*C*) and the real sample value *x* are input first, and then the feature is extracted through the defined five-layer convolution layer, and a residual block structure similar to the generator is added between each convolution layer. Finally, 0 and 1 are used as true and false judgment values to output the discrimination results of the generated samples. The Leaky Relu function is defined as the activation function of all layers, which solves the phenomenon of neuron necrosis in the original activation function, as shown in Figure 3.

### 3.2. Structural Improvement of the C-DCGAN Model

#### 3.2.1. Spectral Normalization

In the generative adversarial networks, the number of parameters increases exponentially with the number of generating layers, which leads to an increase in the amplitude of parameter variation and increases the probability of gradient explosion. By adding spectral normalization to the generator and discriminator respectively [29], the gradient upper bound of the function is restricted to make the function smoother, improve the stability of parameter variation and reduce the probability of gradient explosion.

The relationship between the output and input of the nth convolution layer of C−DCGAN network presented in this paper is shown in Formula (4):

(4) $$x_{n}=D_{n}W_{n}x_{n-1}$$

*D_n_* refers to the diagonal matrix, and *W_n_* is a parameter matrix.

From Formula (4), the output function in C-DCGAN is:

(5) $$f\left(x\right)=D_{n}W_{n}D_{n-1}W_{n-1\ldots \ldots }D_{1}W_{1}X_{0}$$

The upper bound of the gradient of the function is restricted by using the Lipschitz constraint [30]. In Formula (5), the gradient of the function is:

(6) $${\left\|\nabla_{x(f(x))}\right\|}_{2}=\|DnWnD_{n-1}W_{n-1}\ldots D_{1}W_{1}{\|}_{2}\leq {\left\|D_{n}\right\|}_{2}{\left\|W_{n}\right\|}_{2}\ldots {\left\|W_{1}\right\|}_{2}$$

‖*W*‖ is the spectral norm of the *W* matrix, which can also be written as:

(7) $$\|\nabla x(f(x)){\|}_{2}\leq \prod_{i=1}^{N}\sigma (Wi)$$

σ(W) is the maximum singular value of the *W* matrix.

The Formula (7) is normalized as follows:

(8) $${\left\|\nabla x(f(x))\right\|}_{2}={\left\|D_{n}\frac{W_{n}}{\sigma (W_{n})}D_{n-1}\frac{W_{n-1}}{\sigma (W_{n-1})}\ldots D_{1}\frac{W_{1}}{\sigma (W_{1})}\right\|}_{2}\leq \prod_{i=1}^{N}\frac{\sigma (W_{i})}{\sigma (W_{i})}=1$$

The essential process of spectral normalization is SVD layer by layer, which is solved one by one by power iteration because of time constraints and the cost of computing resources.

After the spectral normalization is added, the parameters of the generated network and the discriminated network are shown in the Table 1 and Table 2. 

#### 3.2.2. Loss Function

*KL* divergence or *JS* divergence is usually used to calculate the distance in the original GAN. However, because of the asymmetry of the *KL* divergence, different loss values will be obtained for the same difference value when different calculation methods are used. However, the distance calculated by *JS* divergence is 0 when there is no overlap part in the probability distribution, and over-training will result in a loss value that converges to a fixed value and cannot yield a gradient value.

The *KL* divergence and *JS* divergence are similar to distance *W*. *W* can be understood as the minimum cost under an optimal path planning, and it can be used to compute the distance between two probability distributions. If there is no overlap between the two probability distributions, or the ratio of overlap is small, *W* distance can still measure the distance between the distributions. Therefore, *W* distance can not only provide the distance of distribution, but also guarantee the reliability of gradient information. By defining *W* as the loss function of this experiment, the calculation can, to a large extent, solve the problems of training instability and gradient disappearance.

The *W* distance is defined as follows:

(9) $$W(P_{data},P_{z})=\underset{\gamma -\prod \left(P_{data},P_{z}\right)}{\inf}E(x,y)\sim y[\left\|x-y\right\|]$$

The definition of the *W* distance is derived by a transformation of the given formula, as follows:

(10) $$W(P_{data},P_{z})=\underset{{\left\|f\right\|}_{L}\leq 1}{\sup}E_{x\sim P_{data}}[f(x)]-E_{x\sim p_{z}}[f(x)]$$

It is indicated that the training of the network needs to satisfy the Lipschitz constraint condition, that is ${\left\|D\right\|}_{l}\leq 1$, the amplitude of the parameter change in the training process must be restricted within a certain range.

In this paper, we modify the loss function of the network by adding a gradient penalty term [31], which satisfies the Lipschitz condition. The expression of the gradient penalty term is as follows:

(11) $$H=\lambda E_{x∼P_{\hat{x}}}[{\left(\left\|\nabla_{x}D(\hat{x})\right\|-1\right)}^{2}]$$

The regular term coefficient represents the random sampling value between the real sample value *x* and the generated sample value.

According to the above contents and formulas, the loss functions of the generator and discriminator can be obtained, which are Formulas (12) and (13) respectively:

(12) $$L_{G}=-E_{x\sim P_{z}}[D(G(z|c)|c)]$$

(13) $$L_{D}=E_{x\sim P_{z}}[D(G(z|c)|c)]-E_{x\sim P_{data}}[D(x|c)]+\lambda E_{x\sim P_{\hat{x}}}[{\left(\left\|\nabla_{x}D(\hat{x})\right\|-1\right)}^{2}]$$

### 3.3. Training Process of C-DCGAN

The optimization and improvement points of C-DCGAN have been described in detail in Section 2.1 and Section 2.2 of this paper. Algorithm 1 is the pseudo-code of the improved C-DCGAN algorithm.
**Algorithm 1:** C-DCGAN training processInput: Noise fault data with constraints Output: Enhanced bearing fault sample data1. Initialization generator and discriminator.2. while *i* do:    For step do: Sample mini batch of *n* noise samples from noise *Pz*(*z*)Sample mini batch of *n* examples from data generating distribution *Pdata*(*x*)Add constraints *C* to the generatorNoise sample input generator to obtain generated dataUpdate the discriminator by its stochastic gradient: $\nabla \frac{1}{n}\sum_{i=1}^{n}\left[\log D(x^{\left(i\right)})+\log(1-D(G(z^{\left(i\right)}|c))\right)]$End for3. Sample mini batch of n noise samples from noise *Pz*(*z*)4. Add constraints *C* to the discriminator5. Update the generator by its stochastic gradient:$\nabla \frac{1}{n}\sum_{i=1}^{n}\log(1-D(G(z^{\left(i\right)}|c)))$6. end while   *I* is the maximum number of iterations in the training process. Step is the training times of the discriminator.

### 3.4. One-Dimension Convolutional Neural Network

One-Dimension Convolutional Neural Network (1−D−CNN) is a deep feedback neural network. Since the bearing fault data is one-dimensional data, 1−D−CNN can be used for one-dimensional sequence processing and convolution operations.

Since the convolutional neural network can achieve muti-category classification effects, this paper uses 1−D−CNN to classify the faults of rolling bearings and mixes the original fault data and the generated sample data into the 1−D−CNN network for classification and diagnosis.

In this paper, the bearing fault sample data is input into multiple convolutional layers in the 1−D−CNN model, the input data is convolved through the convolutional layer, and the Relu function is used to activate and add nonlinear features to the feature pool and then use the maximum pooling layer to reduce the input data length of each layer and reduce the probability of over fitting [32]. Finally, the fault data features are processed through the fully connected layer and input to the softmax layer to calculate the probability of fault category and output classification result. The 1−D−CNN is literately trained, and the parameters of the model are literately updated according to the value of the cross-entropy loss function calculated by iteration until the end of the training.

The 1−D−CNN parameters in this paper are shown in Table 3.

### 3.5. Fault Diagnosis Algorithm

**Figure 4:**
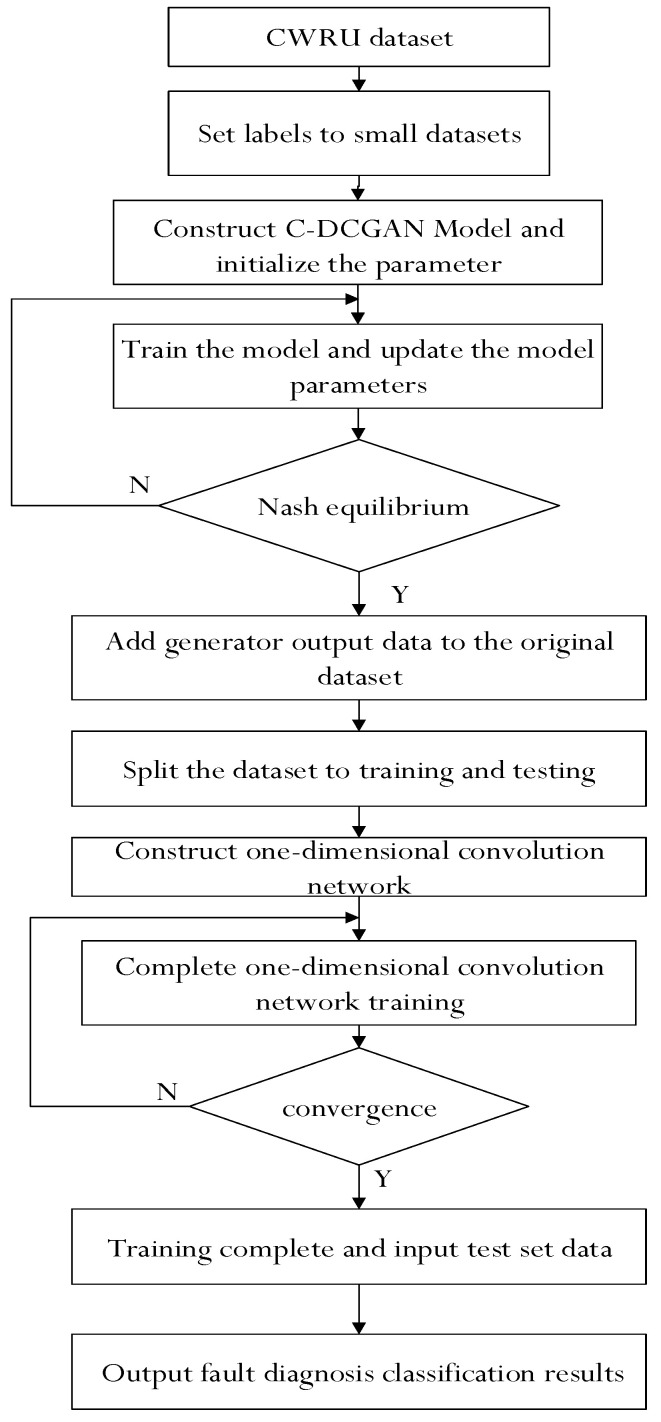
Overall flow chart.

Because the fault diagnosis classification model needs sufficient robust data set to ensure its effectiveness, rich data categories and other characteristics will improve its performance. At the same time, it is necessary to maintain the stability of the training process of the countermeasure generation network. In order to optimize the solutions to the problems mentioned above, a fault diagnosis method based on C−DCGAN network structure is proposed in this paper, and its main flow is shown in Figure 4.

## 4. Experiment and Result Analysis

### 4.1. Data Set

**Figure 5:**
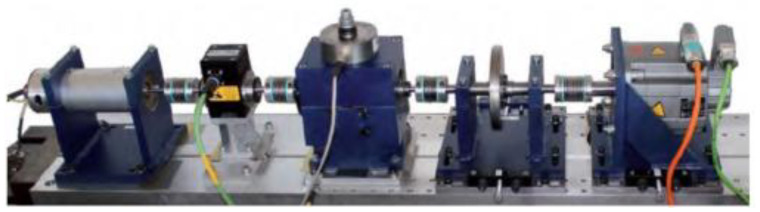
Test stand.

For this paper, the experimental data from the rolling bearing on the inner and outer rings and the rolling body was recorded, after the use of electric spark technology on the rolling bearing had caused different degrees of single-point bearing fault. Figure 5 is the test stand; the diameters of the fault damage were 0.18 mm, 0.36 mm, 0.54 mm, and 0.71 mm, respectively. Then the vibration signals were collected under the conditions of 0HP, 1HP, 2HP and 3HP, respectively; the sampling frequency was 12 kHz.

In this experiment, faults are divided into 10 categories. In order to ensure that the statistical characteristic distribution of fault samples follows that of the distribution of a large number of overall fault characteristics, 1024 consecutive sample points are intercepted on the original vibration signal for each small number of fault samples and a large number of overall fault samples are generated, with a total of three data sets *A*, *B* and *C*. Data set *A* is mainly used to train C−DCGAN to generate high-quality fault sample data, so it mainly contains the original fault sample data of various types of faults in the original fault data set. Data set *B* is mainly used to train the classification model for fault classification and diagnosis. The simulated fault samples generated from data set *A* are mixed into the original data set *A*, and the mixed fault data is used as the training set. Data set *C* is a test set to test the training convergent classification model. The number of experimental samples is shown in Table 4.

### 4.2. Experimental Results and Comparative Analysis

In order to verify the feasibility of the fault diagnosis method based on the C-DCGAN extended data set, the comprehensive indexes of positive case accuracy and negative case accuracy are used to evaluate the method.

“Accuracy” is the ratio of the number of correctly classified failure samples to the total number of failure samples when testing against a failure test set.

“Recall” is the ratio of the number of failure samples from all positive cases to the number of failure samples from the actual positive cases in the correct classification.

“Specificity” refers to the ratio of all false samples to all actual false samples in the predicted accurate fault samples.

G-mean, an index obtained by combining the accuracy of positive cases and negative cases, is usually used to evaluate the classification effect when the data distribution is unbalanced.

(14) $$Accuracy=\frac{TP+TN}{TP+FN+FP+TN}$$

(15) $$Recall=\frac{TP}{TP+FN}$$

(16) $$Specificity=\frac{TN}{TN+FP}$$

(17) $$G-mean=\sqrt{Recall*Specificity}$$

*TP* refers to the number of real samples judged as true sample categories; *TN* refers to the number of false samples judged as false sample categories; *FP* refers to the number of false samples judged as true sample categories, that is, the number of samples that are mistakenly classified; *FN* refers to the number of real samples judged as false sample categories, that is, the number of real samples omitted.

**Figure 6:**
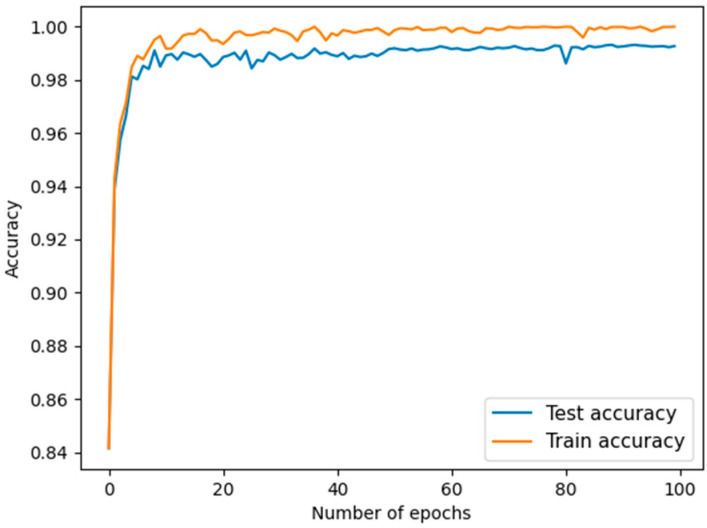
Accuracy.

**Figure 7:**
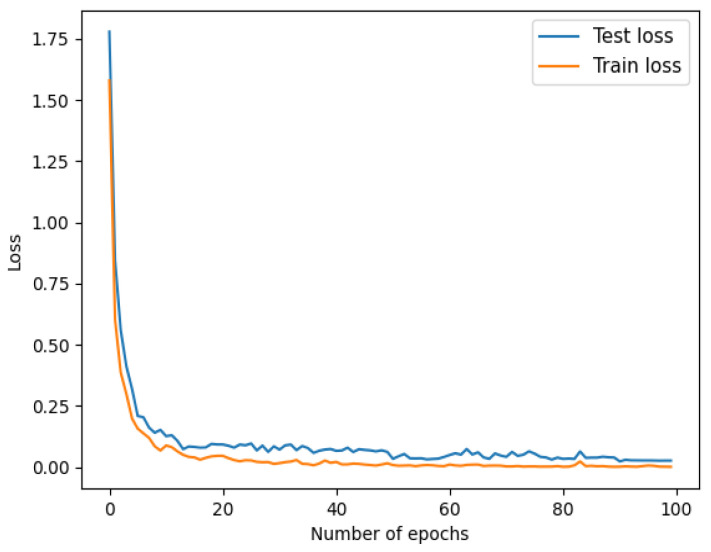
Loss.

The accuracy and the value of loss are shown in Figure 6 and Figure 7.

**Figure 8:**
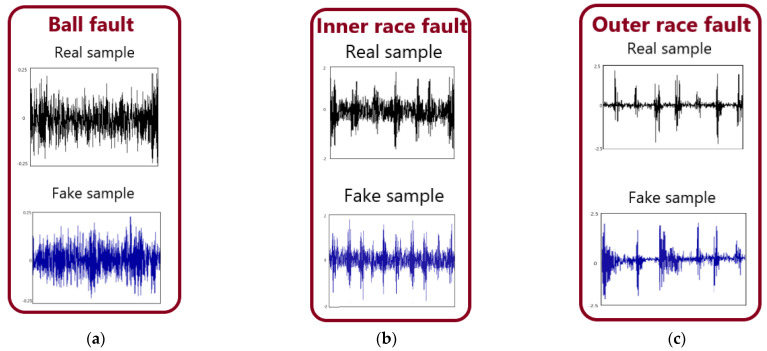
Time domain diagram of the fault.

Figure 8a–c shows the time domain diagrams of the original fault samples of the rolling element, inner ring and outer ring, and the corresponding time domain diagrams of the samples generated by C−DCGAN. It can be seen from Figure 8 that the fault sample data generated by C−DCGAN is not exactly the same as the original data, but its overall distribution is similar to the original sample data. At the same time, the generated data has expanded the diversity of the real sample data, which proves that the generation network can be used to effectively expand the unbalanced data set, so as to solve the problem of data imbalance in the data set. 

**Figure 9:**
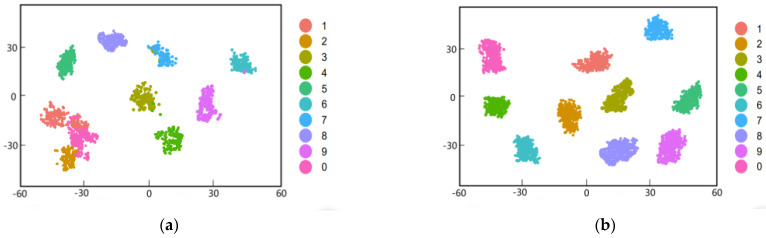
Visual diagram of fault classification: (**a**) CWRU(original)+1-D−CNN; (**b**) proposed method.

As shown in Figure 9, the data set of original unbalanced fault and the expanded fault are used for fault diagnosis by the same classifier, and T−SNE visualization is performed. Figure 9a is the diagnostic classification result of the original unbalanced fault data set. It can be seen that there are many overlapping cases of different types of faults. Figure 9b shows the diagnostic classification results after the data set is balanced and expanded by the generated model structure in this paper. It can be seen that C−DCGAN can generate muti-category fault sample data and mix the generated simulated fault data into the original fault data set. Use of the mixed fault data set for fault diagnosis and classification can improve the clustering effect of each type of fault sample data, and effectively improve the accuracy of fault diagnosis and classification. It can be seen from the classification effect in Figure 9 that the sample values of bearing faults of the same category are gathered together, while the fault data of different categories can be clearly separated according to the fault category, and most of the sample data can be correctly classified.

**Figure 10:**
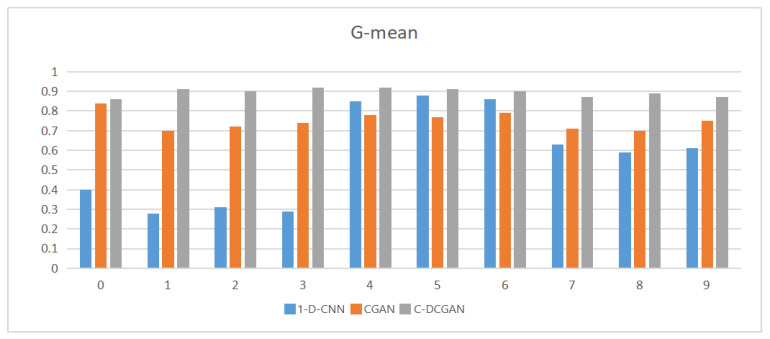
*G*−mean value under ten categories.

According to the definition and formula, the value range of *G*−mean is (0, 1), and values close to 1 indicate that the classification effect of *G*−mean is better. According to Figure 10, the original unbalanced fault data of bearing inputted into the 1−D−CNN network for fault diagnosis and the classification result reveals that most of the values are too small, indicating that the original unbalanced small fault data cannot train 1−D−CNN to a high accuracy rate, so the classification effect is poor. CGAN performs supervised data generation under constraints. The simulated fault sample data generated after the constraint guidance is mixed into the original fault data, and input into the classifier for training. After the classifier is trained to Nash equilibrium, the classification is used. It can be seen from the *G*−mean value that generating valid fault data through CGAN and expanding the data set can reduce the impact of uneven distribution of data samples. The performance of C−DCGAN under ten fault categories is the strongest of the three, and the average value returned exceeds 0.8, which can prove that the network structure described in this paper can better balance the expansion of the original fault sample data set. By balancing the fault data set, the classifier is trained to achieve high-accuracy fault classification and diagnosis, reducing the impact of classification errors caused by uneven data distribution.

**Figure 11:**
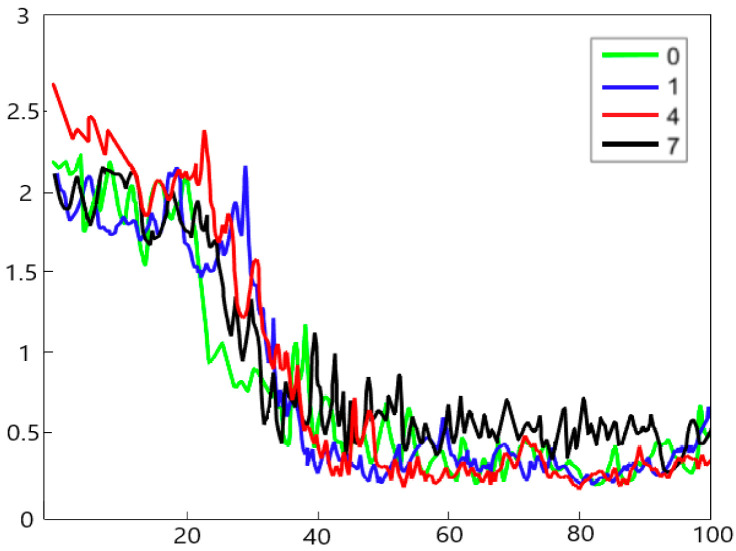
MMD.

In order to verify the validity and authenticity of the fault sample data generated by the model proposed in this paper, the maximum mean discrepancy (MMD) is used for evaluation. This metric evaluates the true rows of the generated simulated sample data by calculating the probability distribution distance between the simulated sample data and the original sample data; the calculation formula is shown in Formula (18):

(18) $$MMD(X,Y)={\left\|\frac{1}{m}\sum_{i=1}^{m}\phi (x_{i})-\frac{1}{n}\sum_{j=1}^{n}\phi (y_{j})\right\|}_{K}^{2}$$

where *K* indicates that the distance between the original and generated data set, and which is mapped to the regenerated Hilbert space by the function of Gaussian kernel Randomly selected 0, 1, 4, and 7 categories of fault data, as shown in Figure 11, in the process of generating data for these four different fault categories, as the number of training iterations increases, the maximum mean difference overall shows a downward trend, and the probability distribution between the original fault data and the generated fault samples gradually decreases. Scaled down, this verifies the authenticity of the simulated fault samples generated by the adversarial generative network proposed in this paper.

**Figure 12:**
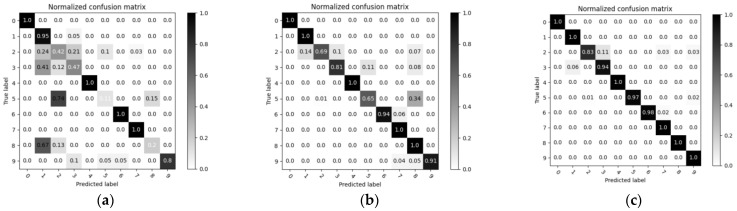
Confusion matrix: (**a**) CGAN; (**b**) C−DCGAN (without SN); and (**c**) C−DCGAN.

In order to verify that the C−DCGAN fault diagnosis model proposed in this paper is suitable for fault diagnosis of bearings in the case of unbalanced sample data, the model is compared with the following two different diagnostic models of adversarial generative networks, with a 1:20 imbalance. The proportion of the fault data set is defined, and the highly unbalanced data set is input into three fault diagnosis models trained to Nash equilibrium, the trained diagnostic model is then used to classify the fault diagnosis of the test set. The classification results are classified into a confusion matrix, as shown in Figure 12. Under the premise of unbalanced sample distribution, the data generation quality of the generative model can be reflected according to the classification accuracy of fault diagnosis. In Figure 12, the fault diagnosis model proposed in this paper evinces high accuracy and can therefore be applied to bearing fault diagnosis scenarios with small sample fault data sets.

In order to further verify that the conditional deep convolutional adversarial generation network proposed in this paper can improve fault diagnosis classification accuracy by balanced expansion of the small sample fault dataset, common fault diagnostic methods, such as C−DCGAN+SVM, C−DCGAN+LSTM, C−DCGAN+1-D−CNN, infoGAN+1−D−CNN, and CGAN+1−D−CNN were adopted for a comparison of rolling bearing fault diagnosis. LSTM is a type of long short-term memory network that detects and classifies faults by learning the temporal information between fault features. C−DCGAN+SVM is a type of process in which, after expanding the small sample data set through the confrontation generation network proposed in this paper, the data set is input to the SVM network for classification training, and the trained classifier is used to perform fault diagnosis and classification on the test set. infoGAN+1-D−CNN is based on an information generation adversarial network for data generation, and it then uses one-dimensional convolutional network for fault feature extraction and fault classification. CGAN is based on GAN and adds constraints for data generation.

According to Table 5, it can be seen that the adversarial generation model proposed in this paper can effectively expand the fault data set and use a variety of classifiers for fault diagnosis, and the classification accuracy can reach 90%. The results show that the fault diagnosis method proposed in this paper can effectively improve the fault diagnosis accuracy of rolling bearings compared with several common fault diagnosis methods based on data augmentation.

## 5. Conclusions

In this paper, a conditional deep convolution generative adversarial network is proposed, one which can effectively expand the data set of small samples, improve the data imbalance of small samples, and improve the precision of bearing fault diagnosis. Compared with other fault diagnosis methods, the demonstrated improved loss functions and network structure can improve the stability of C-DCGAN and reduce the probability of gradient vanishing and gradient explosion. The experimental results show that the fault diagnosis model designed in this paper can effectively improve the classification accuracy of these 10 types of faults and better diagnose the more complex types of faults in the operation of mechanical equipment. However, there are still some remaining concerns. If the fault category is increased or the mechanical operation environment is disturbed, the classification accuracy of the classifier will be reduced to a certain extent, and the model training will be difficult to converge to the ideal state; Future research will aim at the construction of the fault diagnosis model in order to realize multiple fault classifications.

## Figures and Tables

**Table 1:** Network parameters for generator.

Network Layer	Convolution Nucleus	Step Length	Activation Function	Learning Rate	SN
Input	4*4	0	Relu		N
Deconv1	5*5	2	Relu	0.001	Y
Deconv2	5*5	2	Relu	0.001	Y
Deconv3	5*5	2	Relu	0.001	Y
Deconv4	5*5	2	Relu	0.001	Y
Deconv5	5*5	2	Relu	0.001	Y
Output	5*5	2	Tanh		N

**Table 2:** Network parameters of discriminator.

Network Layer	Convolution Nucleus	Step Length	Activation Function	Learning Rate	SN
Input	5*5	2	Leaky Relu		N
Conv1	5*5	2	Leaky Relu	0.001	Y
Conv2	5*5	2	Leaky Relu	0.001	Y
Conv3	5*5	2	Leaky Relu	0.001	Y
Conv4	5*5	2	Leaky Relu	0.001	Y
Conv5	5*5	2	Leaky Relu	0.001	Y
Output	4*4	0	Leaky Relu		N

**Table 3:** Network parameters for 1−D−CNN.

Network Layer	Kernel Count	Kernel Size	Stride	Padding
Conv1	32	1*9	1	1
BN				
Maxpool	32	1*5	2	0
Conv2	64	1*5	1	1
BN				
Maxpool	64	1*5	2	0
Conv3	128	1*5	1	1
BN				
Maxpool	128	1*5	2	0
Conv4	256	1*5	1	1
BN				
Maxpool	256	1*5	2	0
Flatten				
FC1				
FC2				
Softmax				

**Table 4:** Number of experimental samples.

Fault Location	Inner			Outer			Ball			Normal
category	0	1	2	3	4	5	6	7	8	9
diameter	0.18	0.36	0.54	0.18	0.36	0.54	0.18	0.36	0.54	0.00
Data set A	500	500	500	500	500	500	500	500	500	500
Data set B	1500	1500	1500	1500	1500	1500	1500	1500	1500	1500
Data set C	600	600	600	600	600	600	600	600	600	600

**Table 5:** Comparative experimental data.

**Experience**	**Accuracy (%)**	**Standard Deviation**
C-DCGAN+SVM	92.51	±0.75
C-DCGAN+LSTM	97.28	±0.33
C-DCGAN+1-D-CNN	99.01	±0.19
infoGAN+1-D-CNN	98.17	±0.41
CGAN+1-D-CNN	97.82	±0.33

## Data Availability

Not applicable.
